# Electroosmotic Perfusion, External Microdialysis: Simulation and Experiment

**DOI:** 10.1021/acschemneuro.3c00057

**Published:** 2023-06-28

**Authors:** Michael T. Rerick, Jun Chen, Stephen G. Weber

**Affiliations:** Department of Chemistry, University of Pittsburgh, Pittsburgh, Pennsylvania 15260, United States

**Keywords:** Microfluidic, electroosmosis, peptidase, sampling, rate constant

## Abstract

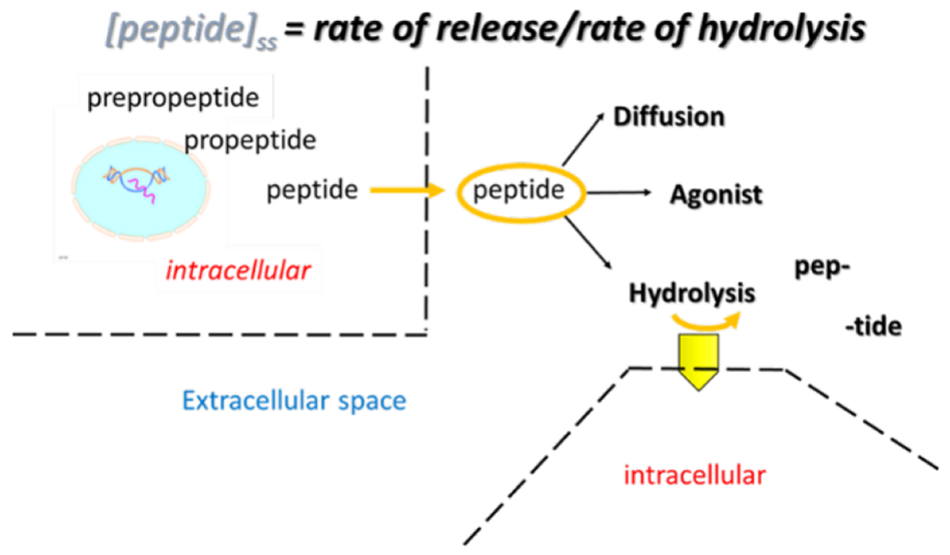

Information about the rates of hydrolysis of neuropeptides by extracellular peptidases can lead to a quantitative understanding of how the steady-state and transient concentrations of neuropeptides are controlled. We have created a small microfluidic device that electroosmotically infuses peptides into, through, and out of the tissue to a microdialysis probe outside the head. The device is created by two-photon polymerization (Nanoscribe). Inferring quantitative estimates of a rate process from the change in concentration of a substrate that has passed through tissue is challenging for two reasons. One is that diffusion is significant, so there is a distribution of peptide substrate residence times in the tissue. This affects the product yield. The other is that there are multiple paths taken by the substrate as it passes through tissue, so there is a distribution of residence times and thus reaction times. Simulation of the process is essential. The simulations presented here imply that a range of first order rate constants of more than 3 orders of magnitude is measurable and that 5–10 min is required to reach a steady state value of product concentration following initiation of substrate infusion. Experiments using a peptidase-resistant d-amino acid pentapeptide, yaGfl, agree with simulations.

## Introduction

Neuropeptides comprise a large and important class of signaling molecules.^1−8^ In the brain, they are generally released from cells and transported in the extracellular space (ECS). Unlike some neurotransmitters which are removed from the ECS by reuptake, neuropeptides are inactivated (or converted to other active forms) by enzymatic action.^9,10^ While knowing the steady-state concentration of a peptide in the ECS is useful, knowing the *rates* of the processes that control a steady state is critical for understanding the extracellular environment. The rate of release controls the flux of peptide into the ECS. The rate of hydrolysis in the ECS controls the tissue volume explored by the released peptide and, with the rate of release, its concentration. The peptide concentration dictates receptor occupancy. Changes in the receptor occupancy by a neuropeptide may result from changes in its release rate or changes in peptidase activity or both.

There are many available approaches to determining enzyme activity, not only peptidases, using a variety of analytical techniques^11^ (Supporting Information, section S1). It is important to use natural substrates in intact tissue or in vivo to infer rates of natural processes and how those might change under various conditions. For example, the membrane-bound enzyme (ectoenzyme) aminopeptidase A (EC 3.4.11.7) converts angiotensin II (Ang II) to Ang III in the ECS. Ang III is converted to Ang IV by aminopeptidase N (EC 3.4.11.2). Aminopeptidase A is inhibited by Ang IV.^12^ Thus, aminopeptidase A’s activity depends on the local activity of aminopeptidase N. Similarly, insulin-regulated aminopeptidase (IRAP, EC 3.4.11.3) is also inhibited by Ang IV;^13^ thus, the activity of one ectopeptidase, aminopeptidase N, producing Ang IV, may influence another, IRAP, nearby. It is difficult to envision how these systems could be investigated adequately without using natural peptide substrates in experimental studies of functional tissue.

It should be possible to infer peptidase activity, at least qualitatively if not quantitatively, by passing a substrate through tissue and determining substrate loss (or product gain) caused by hydrolysis. Indeed, several studies exist. Microdialysis has been used to study peptide hydrolysis by enzymes in vivo in the brain based on retrodialysis of the substrate peptide^14−23^ (Supporting Information, section S2). Another approach is to infuse the substrate near the microdialysis probe with an infusion cannula near the microdialysis probe (Microbiotech, Eicom, and BASi). One study^24^ compared the cannula/probe method with the retrodialysis approach. The cannula/probe failed because all substrate infused was hydrolyzed. Thus, only very slow reactions are accessible with these devices. In addition, the diameter of these devices at ∼350 μm inhibits application to small brain regions and creates significant insertion trauma.^25−28^ Iontophoresis has been used sparingly for analogous work. Qualitative evaluation of peptide hydrolysis following iontophoretic delivery *into skin* has been investigated for Tyr-Phe,^29^ LHRH,^30^ and delta sleep-inducing peptide.^31^ Felix and Harding used iontophoresis *in brain* to deduce that enzymatic hydrolysis of Ang II to product Ang III increased the overall potency of Ang II.^32^ Low-flow push–pull perfusion (PPP)^33^ has been used to identify different nitric oxide synthase subtypes in rat retina based on measuring nitrate concentration in the presence and absence of specific inhibitors.^34^ Low-flow PPP can identify contents of single cells by direct connection of the “pull” side of the device to the inlet of a nanospray source of a mass spectrometer.^35^ These studies did not determine reaction rates.

Following our discovery that brain tissue has a significant ζ-potential,^36^ we learned how to use this property of brain tissue to infuse solutes into brain and organotypic hippocampal slice cultures (OHSCs) using controlled electrical current^37,38^ and to infuse substrates of known ectoenzymes and determine information about these enzymes in OHSCs^39−43^ and in vivo.^44^ For the in vivo work, direct laser writing by two-photon polymerization was used to manufacture a device (Supporting Information, section S3). A pointed, narrow capillary adjacent to a microdialysis probe was the source of either of two solutions, e.g., artificial cerebrospinal fluid (aCSF) or aCSF with a substrate. Electroosmosis carried the solution out of the capillary, through tissue toward a microdialysis probe in the tissue. Precise control of substrate/inhibitor perfusion at low flow rates (∼10–50 nL/min typically) was achieved using constant current control of electroosmotic flow rate. This in turn controlled the substrate’s residence time in the tissue. Collection by microdialysis, while it diluted the sample, provided manageable sample volumes for online analysis. Combined with online isotopic labeling/capillary LC–MS^2^ detection, quantitative estimates of substrate-to-product conversions were obtained for leu-enkephalin (YGGFL) hydrolysis in vivo. Through the perfusion of HFI-419,^45^ a selective inhibitor of insulin regulated aminopeptidase (IRAP), dose-dependent inhibition of YGGFL hydrolysis was observed in rat neocortex. We concluded that IRAP was not the dominant enkephalin-degrading enzyme in this region.^44^

It occurred to us that direct laser writing could facilitate a significant modification of this device: Why not place the microdialysis probe outside the tissue? The microdialysis probe can be placed in a chamber that is part of the object created with direct laser writing. This would significantly reduce the size of the object placed in the tissue, reducing trauma and the effects of the tissue response on the mass transport properties of the probe. By causing the collected fluid to flow past the microdialysis membrane in a confined volume, collection efficiency may increase and may become more reproducible. Having the microdialysis probe sitting in a well rather than in the tissue would allow the investigator to change the microdialysis probe from a low molecular weight cutoff (MWCO) to a larger MWCO in the same animal/brain region. Thus, there appear to be several advantages to having the microdialysis probe outside of the tissue being studied. We call this arrangement electroosmotic perfusion-external microdialysis (EOP-EMD). Here, we describe simulations of EOP-EMD to investigate the range of chemical reaction rates that can be investigated using this novel device and technique. We further provide preliminary experimental data indicating that the simulations are fairly accurate.

## Results and Discussion

**Figure 1:**
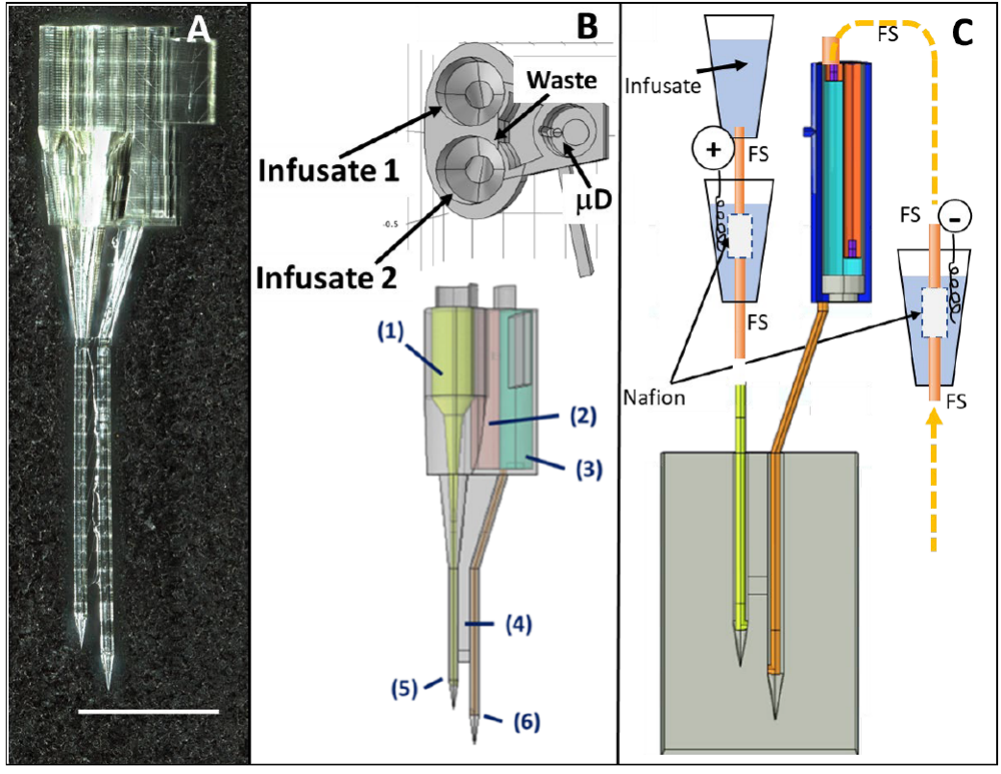
(A) EOP-EMD device. Scale bar is 1 mm. (B) COMSOL model of a device. At the top, “Infusate 1” and “Infusate 2” indicate wells that accept the fused silica capillaries through which the infusates pass from a reservoir to the tissue. There is a third well for the microdialysis probe (μD). The bottom image shows (1) where the capillary for “Infusate 2” sits, (2) overflow reservoir, (3) microdialysis probe, and (4) structural support for the source (5) and sink (6) conduits. (C) The vertically aligned truncated cones are vessels (microfuge tubes) containing physiological electrolyte solution (blue). The upper left pair, one containing infusate and one as the current source, are connected by a fused silica capillary (FS). “+” indicates positive current passing through the coiled silver wire, electrolyte, and Nafion tubing creating electroosmotic flow. The central portion is a 2D slice from the 3D COMSOL model with the tissue (gray), source capillary (yellow), sink capillary (orange), and microdialysis chamber (upper right). Dark blue is fluid outside the probe, and cyan is fluid inside the probe. Capillaries in the probe are orange. Fluid collected by microdialysis goes to the analysis instruments passing through a current sink. The portion of the device where captured solutes are delivered to the microdialysis probe is discussed below and shown in detail in Figure 4.

Figure 1 describes the physical objects used for perfusing tissue with a peptide substrate, collecting product(s), and analysis. Figure 1A,B shows the physical properties of the device. Note that the distal ends of the “legs”, Figure 1B, 5 and 6, are only 90 μm in diameter with a 50 μm inside diameter channel. Source and sink probes are offset to minimize the potential effects of tissue trauma on the measurements. Figure 1C describes how EOP-EMD works. The pair of truncated cones at the upper left of Figure 1C represents the means to electroosmotically deliver a peptide substrate to tissue. This will be called the “infusate delivery system”. The bottom portion of the infusate delivery system is a current interface where current is introduced to create electroosmotic flow. In practice, there would be an infusate delivery system for both infusate channels, but only one is shown here. The central portion of Figure 1C is a snapshot of the 3D model used in the simulation. At the right is the current interface, where current leaves the microfluidic path.

To use a device, the capillaries at the base of the infusate delivery systems are placed in the source ports (not shown in Figure 1C) and a microdialysis probe is placed into its chamber. Prior to this, syringes are used to fill the fluidic channels in the device with physiological electrolyte. Infusate is electroosmotically pumped due to current flowing within the fluidic path of the device. The electrical current from a current source creates ionic current in solution which creates electroosmotic flow. (Details are in ref (44).) Current and fluid (infusate) pass through the tissue and into the chamber (Figure 1B, “μD”) containing a microdialysis probe. Solutes and current pass through the microdialysis membrane. Solutes pass to the analytical system, while current passes out of the fluidic stream to a negative electrode. Two power supplies are used to provide equal and opposite currents so that the animal is at or near the ground potential.

A standard laboratory measurement of the rate of an enzyme-catalyzed reaction involves observing changes in substrate and/or product over time and then using the Michaelis–Menten equation to determine kinetic parameters. The conditions are often such that the substrate concentration is assumed to be constant. Here, these conditions do not apply. Substrate is infused, is diluted by diffusion, and reacts. Substrate/products are collected, and their concentrations are measured. All infused solutions contain an unhydrolyzable leu-enkephalin analog, yaGfl (the lower case implies a d-amino acid), as a standard to which substrate and product concentrations are ratioed. It acts as a surrogate for YGGFL’s mass transport behavior in the absence of its hydrolysis. To infer a rate requires knowing the initial substrate concentration, the final concentration, and the reaction time. Only the final concentration is known by measurement. Simulations help to interpret results quantitatively.

### Simulations

We used COMSOL to create the three-dimensional model of the EOP-EMD probe as input into the Nanoscribe two-photon polymerization instrument as well as to carry out the simulations. (Dimensions of the components are in Supporting Information, section S4). We have carried out simulations of a similar experiment, electroosmotic push–pull perfusion.^46^ The three-dimensional EOP-EMD device was created in COMSOL (as shown in Figure 1B). Simulations include a volume of tissue (1.67 mm vertical (*z*), 1 mm horizontal in the plane shown in Figure 1 (*x*), 0.6 mm horizontal in the remaining plane (*y*)) with the porosity (α = 0.2) and tortuosity (λ = 1.61) of adult male rat cortex.^47^ The simulations comprised three steps: (1) Calculate the electric field given a certain current. (2) Calculate the fluid velocity. (3) Calculate the mass transport and chemical reactions of peptides infused.

In a typical simulation, two solutes were infused from one of the source channels, namely, leucine enkephalin, YGGFL, and yaGfl. A chemical reaction, the enzyme-catalyzed hydrolysis of YGGFL to GGFL, was simulated to investigate the range of reaction rates accessible when using EOP-EMD. All simulated processes are linear: The fluids are not compressible, the viscosity is independent of fluid velocity, the brain tissue is not compressible,^48^ and the rate of substrate hydrolysis is independent of substrate concentration. To the latter point, measurements of enkephalin concentrations in brain in vivo indicate levels in the low picomolar range.^18,49−51^ Experimentally the concentrations of peptides infused are 1 μM with nanoLC and electrospray mass spectrometry for determination of peptides.^44^ Common ectopeptidase Michaelis constants are in the ∼20–200 μM level (e.g., refs (43) and (52−57)). Because the initial substrate concentration *S*_*i*_ ≪ *K*_m_, a linear rate law is used.

### Mass Transport

**Figure 2:**
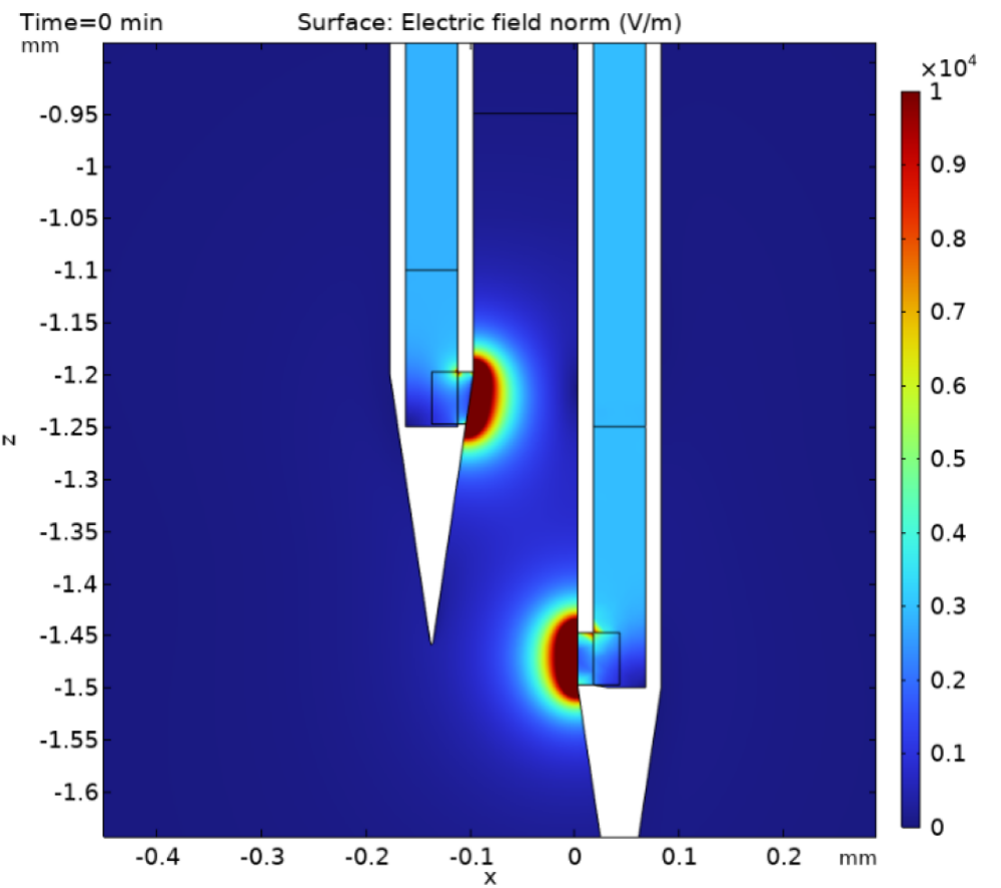
Simulated electric field with 10 μA of current. Numerical scales for horizontal, *x*, and vertical, *y*, are in mm. The scale on the right indicates the electric field. Note that the scale is truncated at 10^4^ V/m to improve visualization of the field magnitude.

Figure 2 shows a color plot of the simulated electric field when applying a current of 10 μA. (Numerical values of parameters used in the simulations are in Table 1). The simulations are three-dimensional, but the image is one plane from that three-dimensional simulation. The field within the 50 μm diameter lumens of the source (left) and sink (right) probes is about 3000 V/m or 3 V/mm. Within the tissue, the field in the tissue near the source and sink orifices increases because the tissue conductivity is lower than the solution conductivity by the ratio of tissue porosity to tortuosity squared, α/λ^2^. Lower conductivity dictates a higher voltage drop to maintain a constant current. Beyond the 20–30 μm adjacent to the source/sink orifices and despite the lower conductivity in the tissue, the field becomes quite low between the source and sink because the current density decreases as the distance from the orifices increases.

**Table 1:** List of Parameters Used in Simulations

parameter	medium	magnitude	comments
ζ potential	Tissue	–0.0228 V	Guy et al.^58,59^
	Fluidic channels	–0.0465 V	Ou et al.;^61^ Kirby and Hasselbrink^60^
Temperature	Tissue	37 °C	
	Our lab	22 °C	
Viscosity of saline solution	Tissue	0.00070 Pa s	Kestin et al.^62^
	Our lab	0.00096 Pa s	Kestin et al.^62^
Conductivity	Tissue	0.139 S/m	McKleskey et al.^63^ plus solution viscosity, porosity, and tortuosity in this table
Conductivity	Membrane	0.046 S/m	McKleskey et al.^63^ plus solution viscosity, porosity, and tortuosity
Conductivity,	Infused solution, body T	1.80 S/m	McKleskey et al.^63^ and solution viscosity
Conductivity	Infused solution, lab T	1.36 S/m	McKleskey et al.^63^ and solution viscosity
Porosity	Tissue	0.2	Sykova and Nicholson^47^
	13 kDa MD membrane	0.2	
Tortuosity	Tissue	1.61	Sykova and Nicholson^47^
	13 kDa MD membrane	2.42	Our unpublished determination
Permeability	Tissue	1.0 × 10^–15^ m^2^	Støverud et al.^44,48^
	13 kDa MD membrane	2 × 10^–19^ m^2^	Kiritsis^64^
Diffusion coefficients	YGGFL, 37 °C	5.5 × 10^–10^ m^2^/s	Li and Carr; Scheibel; Häckel et al.^65−67^
	GGFL, 37 °C	6.5 × 10^–10^ m^2^/s	″
	yaGfl, 37 °C	5.4 × 10^–10^ m^2^/s	″
	YGGFL, 22 °C	4.2 × 10^–10^ m^2^/s	″
	GGFL, 22 °C	5.0 × 10^–10^ m^2^/s	″
	yaGfl, 22 °C	4.2 × 10^–10^ m^2^/s	″

Recall that we determined the ζ-potential of the tissue (about −23 mV) and that of a fused silica capillary (about −45 mV). We have not measured the ζ-potentials of the source and sink conduits (5 and 6 in Figure 1B) that are created with the Nanoscribe. Simulations using either the fused silica ζ-potential or a zero ζ-potential for the source and sink conduits reveal that the fluid flow rate/current ratio in tissue is minimally affected (less than 1%) by the stated change in ζ-potentials of the conduits. In the simulations we use the fused silica ζ-potential. Current through the source capillaries (with the more negative ζ-potential) creates a positive pressure between the device and the tissue (with the less negative ζ-potential) at the source and a negative pressure at the sink. This aids the flow, but the aid is not significant. A simulation of the experiment with 30 μA current reveals a pressure drop between the source and sink orifice, in other words within the tissue, of 14.2 Pa (Supporting Information section S5). The average fluid velocity resulting from this pressure is 7.5 × 10^–8^ m/s. The distance between source and sink orifices is 269 μm, so this velocity would carry solute from source orifice to sink orifice in 60 min. In contrast, the times for solute to travel over the same distance in simulations (which include electroosmotic flow) are considerably smaller (Table 2 and discussed below); thus, pressure plays a small role in mass transport.

**Table 2:** Effect of Current on Operational Parameters in EOP-EMD (Simulated).

		*m*_1_, min	*t*_95_, min
μA	Pé	source to sink	source to μD exit
5	3.0	1.8	14
10	3.9	1.4	9.7
15	4.6	1.1	7.8
30	6.7	0.78	4.0

**Figure 3:**
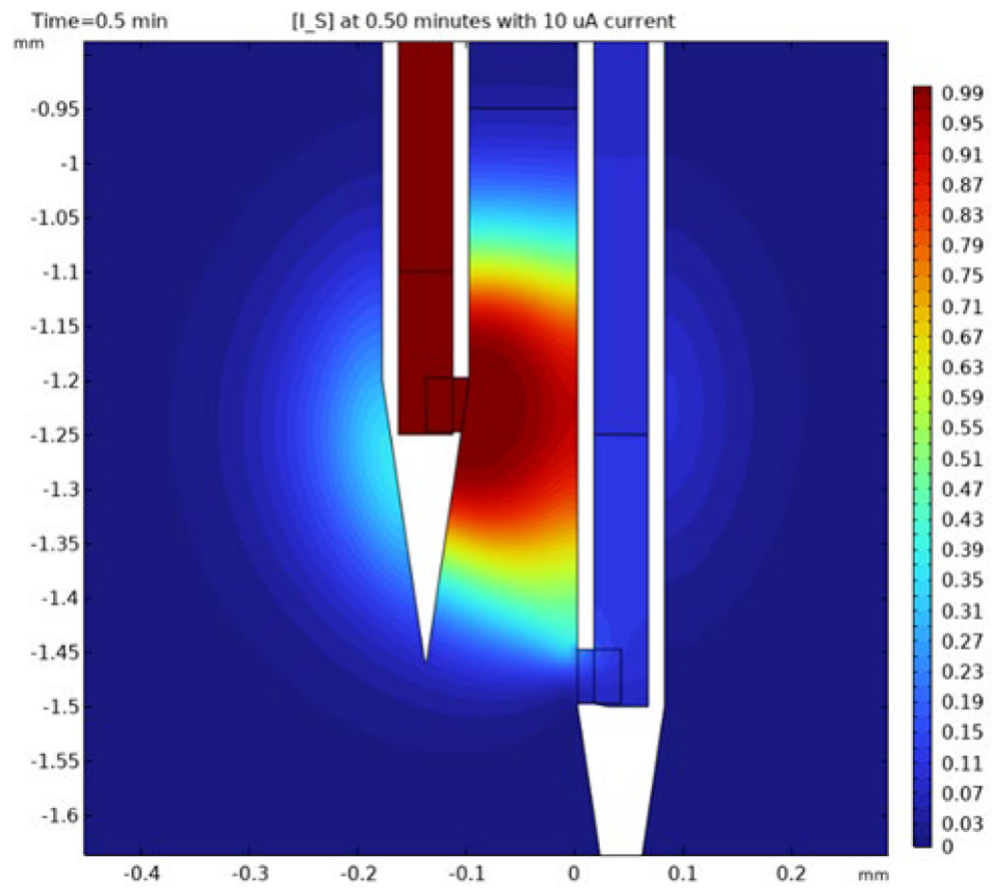
Simulated concentration profile of yaGfl 30 s after applying 10 μA. Peptide emanates from the left, source, orifice and leaves the tissue at the sink orifice on the right.

Figure 3 shows the simulated concentration of yaGfl within the tissue 30 s after initiating current flow (10 μA). It is immediately obvious that diffusion is very influential. This is reflected quantitatively in the Péclet (Pé) number, a measure of the relative influence of a deterministic velocity to that of diffusion over a specific distance (Table 2). The values of Pé are greater than unity but not by much. Table 2 also shows the average residence time, *m*_1_, of an unreactive substrate in the tissue (source to sink). The average residence time is the first moment of the observed distribution of arrival times following a pulse-input of solute yaGfl (delta function). Our experiments, and thus the simulations, use a step, not a pulse, input of solute. The derivative of a step function is a delta function, so we differentiated our simulated tissue concentration vs time curves to obtain the first moment, or average residence time, of the solute.^68^ (See Supporting Information section S6 for the distributions.) The times are fairly short, less than 2 min.

**Figure 4:**
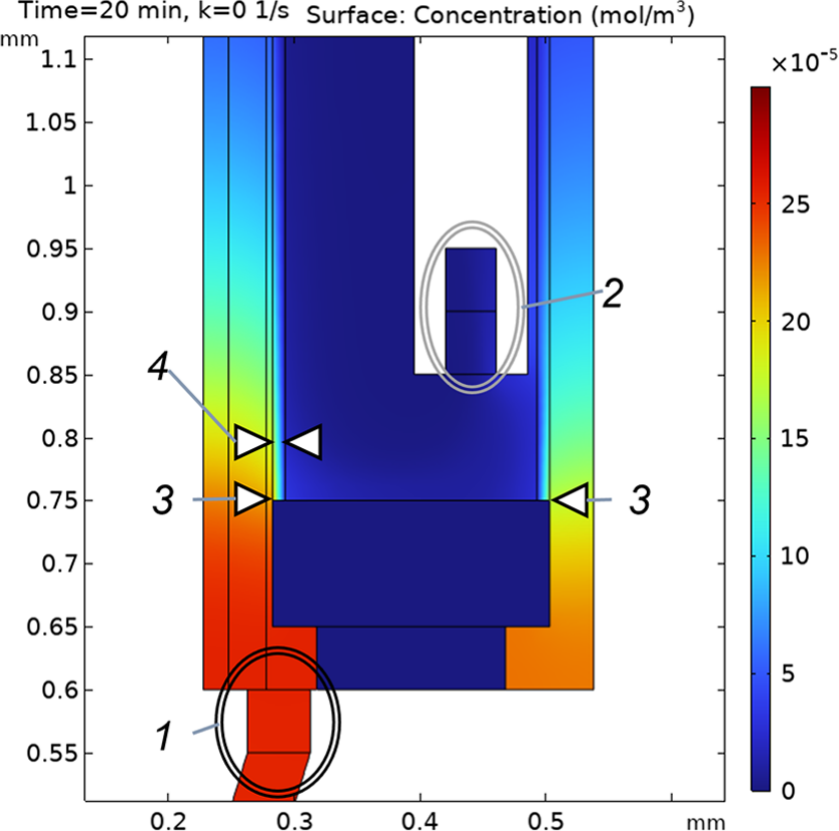
Pictured is the portion of the microdialysis chamber indicated in Figure 1C. It shows the simulated steady-state profile of yaGfl concentration after applying 10 μA. Peptide enters the chamber at 1 and leaves in the dialysate flow at 2. Item 3 indicates the top of the “glue plug”/bottom of the membrane, 4.

Figure 4 shows the simulated concentration of yaGfl in the microdialysis chamber (recall Figure 1C) after 20 min (97% of steady state) with a current of 10 μA. Captured fluid is directed (“1” in the figure) into the base of the chamber containing the microdialysis probe. Microdialysate containing peptides exits the lumen of the probe at 2. (The upper surface of the “glue plug” defining the bottom of the dialysate fluid probe is at 3.) The peptide may diffuse through the membrane (4) or exit to waste (not shown; see Supporting Information section S7). Microdialysis creates a clean sample, simplifying quantitation of peptide solutes. However, qualitatively seen in the figure, the concentration inside the probe is lower than that entering the chamber. A significant factor dictating the magnitude of the dilution is the difference in flow rates; the microdialysate flow rate is 500 nL/min while the electroosmostic flow rate, at approximately 0.8 nL/min per μA (Supporting Information, section S8) at body temperature, is only about 8 nL/min under conditions in the figure.

What we portray here as a residence time of yaGfl becomes a reaction time for YGGFL. The distribution of times would seem to be problematic for the determination of reaction rates in tissue. However, a distribution of residence times like those here were observed in a similar experiment, electroosmotic push–pull perfusion in organotypic hippocampal slice cultures.^46^ We found that there was essentially no difference in the parameters, *K*_m_ and *V*_max_, determined from the data when using the entire residence time distribution and when using only the first moment (average) residence time.^46^ Thus, the wide distribution does not prevent quantitative estimates of rate parameters from data.

### Peptide Hydrolysis

We carried out simulations over a range of first-order rate constants for the hydrolysis of YGGFL to GGFL and Y. Each simulation tracked the concentrations of YGGFL, GGFL, and yaGfl over time up to 40 min. The choice of 40 min is somewhat arbitrary. The computations are time-consuming, so shorter times are advantageous, but longer times bring the system closer to a steady state. The “times to 95% of steady state” in Table 2 show that 40 min is significantly greater than those times.

**Figure 5:**
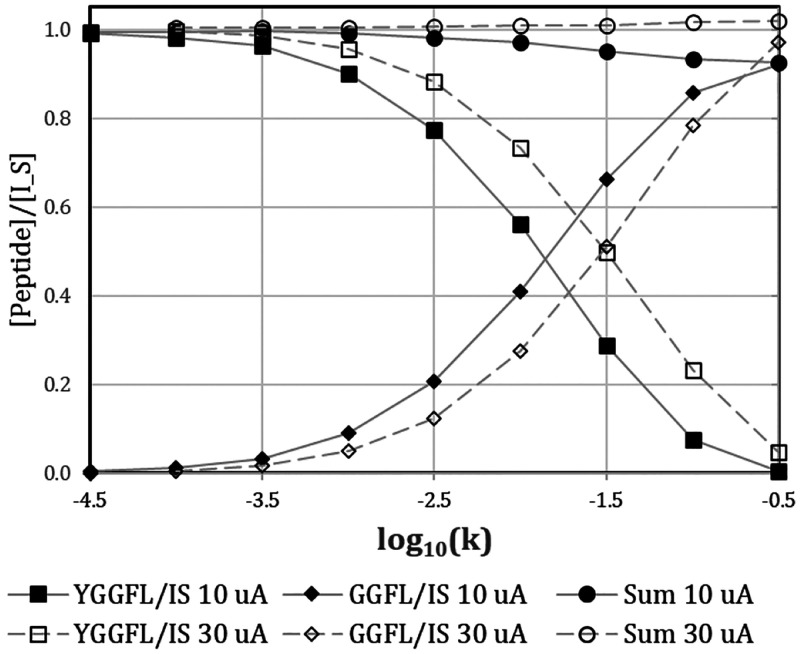
Simulated ratios of YGGFL and GGFL concentrations to the internal standard (I_S, yaGfl) in microdialysate exiting the probe. Note that the curves do not have exactly the same shape due to the small differences among the diffusion coefficients of the three solutes.

Figure 5 illustrates the simulated substrate and product concentrations for hydrolysis of YGGFL with product GGFL for two currents, 10 and 30 μA, promoting flow. Qualitatively, it is obvious that higher current will lead to shorter reaction times; thus, higher current improves the ability to measure faster rates, while lower currents are more effective for slower rates. However, the effect is not linear due to the significance of diffusion. Tripling the current increases the sensitivity to rates by about a factor of only 2.5 as judged by the horizontal distance between the points where each YGGFL/GGFL pair of curves cross. In contrast, the range of rate constants available with a single current is quite wide, probably 10^3^ or more. This dynamic range is governed by the detection limit of the minority species because measurement uncertainty typically scales inversely with the magnitude of the measured concentration. For example, a slow rate that creates 5 nM product from 1000 nM substrate should be assessed from the product concentration because the change of 5 nM out of 1000 nM substrate will be difficult to quantitate. Detection limits for enkephalins in rat brain with microdialysis/LC/MS^2^ are in the single-digit pM range.^50,51^ Thus, measuring the change from 0 to 5 nM product is manageable. The dynamic range can be controlled. Decreasing (improving) detection limits, tuning reaction times (lower for high rate constants and vice versa), and increasing substrate concentration for fast reactions all widen the accessible range of rates for a given current.

Our earlier work^43^ in hippocampal tissue cultures provides a data point for assessing the relevance of the range of accessible rates constants displayed in Figure 5. We determined the aminopeptidase N activity in OHSCs. In round numbers log_10_(*k*) was about −1.3 (*k* ∼ 0.05 s^–1^). This activity would clearly be measurable with EOP-EMD at the currents used in Figure 5.

**Figure 6:**
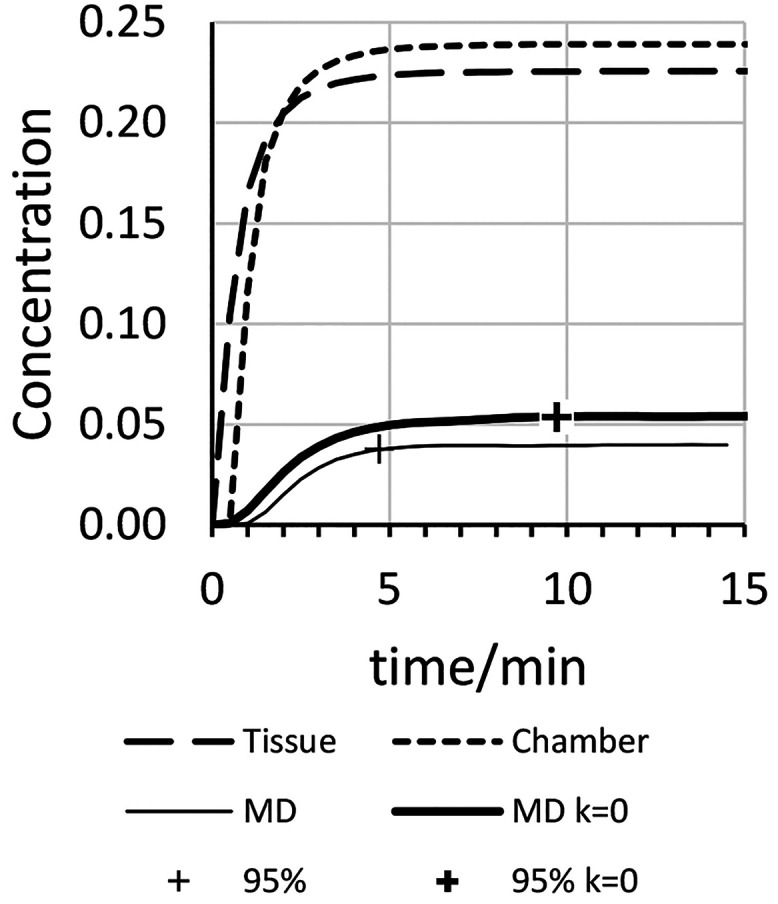
YGGFL hydrolysis rate is *k* = 0.005 s^–1^ except for the bold curve. Current is 10 μA. Plots have concentration of substrate YGGFL at the sink orifice (“Tissue”), at the entrance to the microdialysis chamber (short dash:, “Chamber”), and at the entrance to the capillary carrying fluid out of the microdialysis probe (lighter solid, “MD”). The fourth, bold, curve is as the “MD” curve but with *k* = 0. The “+” symbol in the latter two curves indicates where the concentration is 95% of that at 40 min. Note that concentrations labeled “MD” have been multiplied by 10 for ease of visualization.

The device, as designed, has a fluidic channel passing from the tissue to the microdialysis chamber. Solutes passing into the microdialysis chamber are swept away for quantitative analysis. These mass transport processes affect the response time of the measurements and the concentrations of sought-for compounds. Figure 6 shows the simulated concentration of YGGFL vs time at three points in the passage of solute through the device, namely, leaving the tissue (see Figure 2), entering the microdialysis chamber (see Figure 4, item 1), and leaving the lumen of the microdialysis probe (see Figure 4, item 2). Note that the time resolution in Figure 6 is 0.5 min, so the shapes of the dashed curves do not accurately show the sigmoidal shape expected at short times. The major conclusion from Figure 6 is that microdialysis adds a significant time delay as well as decreasing the measured concentration. (Note the difference in scales for the microdialysis curves vs the others.) For the *k* = 0 curve (10 μA), the simulation gives a recovery ratio (concentration detected/concentration infused) of 5.5 × 10^–3^. It would be beneficial where possible to make measurements of solution at the point indicated in Figure 4 (item 1) avoiding further sample transport and dilution. Of course, microdialysis has advantages, namely, removing the analyte from high molecular weight species in the sample and increasing the velocity of the sample’s transport to a measurement system.

**Figure 7:**
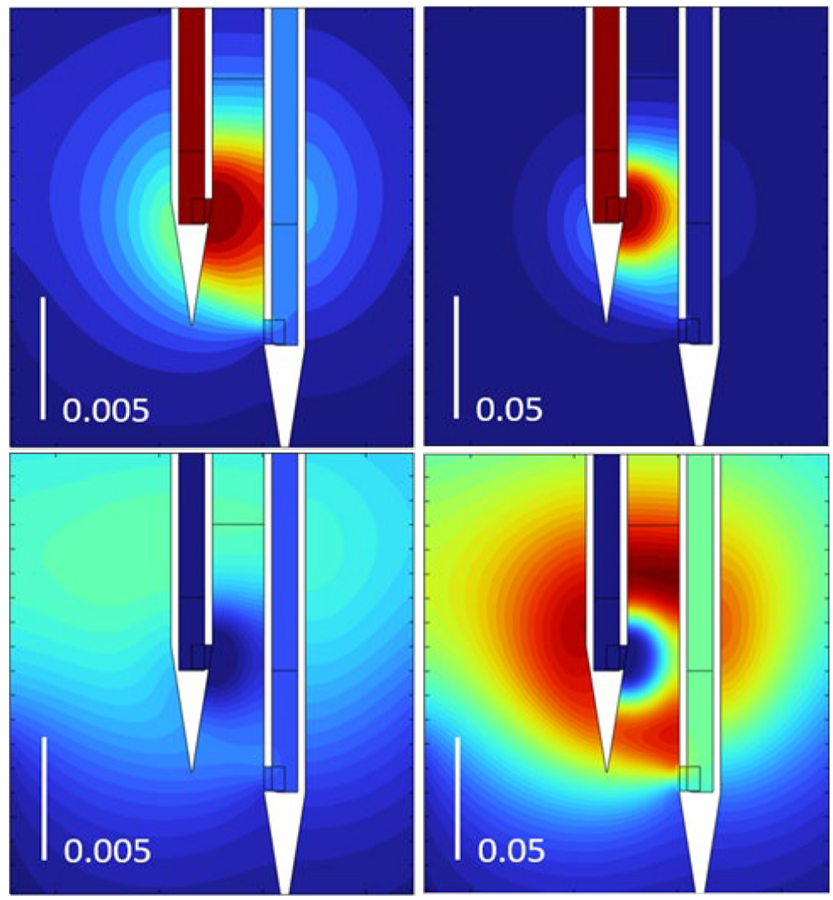
Simulated concentration of YGGFL (top) and GGFL (bottom) for two values of the hydrolysis rate constant, 0.005 and 0.05 s^–1^. The vertical bar is 250 μm. In the substrate pair (top), the dark red/brown is the maximum of the color scale at 1 mol/m^3^, while for product (bottom) it is half that. The color scale goes from dark red to blue in the same order as the visible spectrum.

Figure 6 has a fourth curve representing the simulated concentration of YGGFL leaving the lumen of the microdialysis probe (Figure 4, item 2) under conditions where *k* = 0 s^–1^. Aside from the expected higher concentration than the corresponding curve with *k* = 0.005 s^–1^, we note that the “zero *k*” curve reaches 95% of steady state (“+”) more than twice as slowly as the curve with a reaction. We speculate that this is again an effect related to diffusion. Solute YGGFL escaping by diffusion from the path leading to the sink orifice is consumed by the reaction when *k* is not equal to zero. This creates a quasi-steady-state concentration profile of YGGFL. Without the reaction, the concentration profile continues to evolve for a longer time. This leads to a softer increase in YGGFL concentration with increasing time after initiating infusion of the peptide when there is no reaction consuming the YGGFL. Figure 7 shows simulated concentration profiles of YGGFL and GGFL in tissue for two rate constants. The images support the notion that the region of tissue most influential in the measurements is between the source and sink orifices.

**Figure 8:**
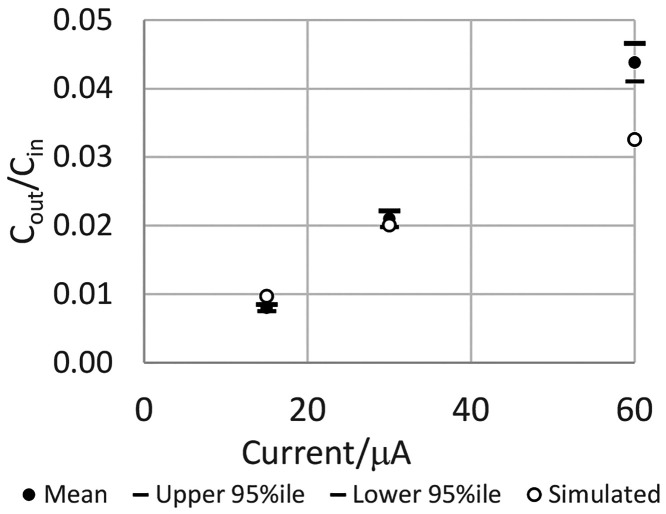
Ratio of experimentally measured yaGfl concentrations to the concentration in the source, *C*_out_/*C*_in_. Open circles are calculated, and closed circles are experimental in vivo. 95% CI is indicated by horizontal bars.

To establish the validity of the mass transport aspects of the simulation, we carried out in vivo measurements in an anesthetized male adult Sprague-Dawley rat. We determined the recovery of infused yaGfl at three currents, namely, 15, 30, and 60 μA. Figure 8 demonstrates agreement of experiment and simulation at 15 and 30 μA but not at 60 μA. (For raw data see SI section S9). Thus, we have not carried out simulations at currents greater than 30 μA.

## Conclusions

Current-induced flow facilitates measurements using microdialysis without placing the microdialysis probe in the tissue. In addition, there are many advantages to using a low, electrically controlled flow of physiological saline to perfuse tissue and cause the perfusate to flow past a microdialysis probe outside the tissue. We earlier showed quantitatively in organotypic hippocampal cultures^61^ that hydrolysis rates of YGGFL, which is neuroprotective in the hippocampus, were significantly different in two regions of the hippocampus. This demonstrates that there is merit to pursuing a path for measuring peptidase rates with natural substrates in tissue cultures. However, having the ability to do such experiments in vivo would provide a wealth of information on the dynamics of peptide-mediated signaling. While we demonstrated a step in that direction with electroosmotic perfusion-microdialysis^44^ (with the microdialysis probe in the tissue), having the microdialysis probe outside the head has many advantages in principle. The (unproven) potential advantages are less tissue trauma, less membrane fouling, and less reliance on retrodialysis^69^ to introduce substrates.

The simulations help to define the experimental conditions. Most clearly, the delay between introduction of a substrate and reaching near-steady-state concentrations is known from the simulations. It seems sensible to collect fractions or inject samples (depending on the analytical methods) every 5–10 min. The time for a few replicates with and without an inhibitor, for example, is then about an hour. The model also shows, Figure 6, that eliminating the microdialysis probe has advantages in measurement time and sensitivity but obviously also has the disadvantage of having a more complex sample to analyze. One solution to this disadvantage is to create a membrane in the sink channel of the device itself as carried out by Song et al.^70^

While our focus is on studying rates of extracellular hydrolysis of peptides in the brain, EOP-EMD could be used to capture solutes native to the ECS of tissue, effectively improving on ordinary microdialysis by having a smaller diameter (90 vs 220 μm), having a pointed tip to minimize insertion trauma, and relying less on passive diffusion to collect ECS fluid. Of course, here we have emphasized using the device for understanding rates of peptide hydrolysis.

## Experimental Section

### Simulations

The finite element model constructed for both probe designs follows our previously published models.^38,71^ We used COMSOL 6.1’s “Electric Current”, “Free and Porous Media Flow”, and “Transport of Diluted Species in Porous Media” modules to calculate the electric field, the fluid flow rate, and the mass transport and chemical reactions. Parameters that influence the mass transport are shown in Table 1.

The microdialysis membrane was modeled as a homogeneous porous matrix, and the microdialysis inlet was set to fully developed flow of 8.33 × 10^–12^ m^3^/s (0.5 μL/min). The microdialysis probe outlet capillary had a length of 0.1 mm, much shorter than the actual dimensions. The effect of the “missing” capillaries on flow was modeled with a pressure boundary condition at the end of the 0.1 mm capillary. The pressure at this boundary is the backpressure generated across the missing capillary length at a flow rate of 0.5 μL/min which can be calculated using the Hagen–Poiseuille equation. There are three capillaries in series between the MD probe outlet and the sample loop exit which comprise the “missing” capillaries. Their dimensions are 50 mm × 0.04 mm, 1000 mm × 0.1 mm, and 140 mm × 0.1 mm, yielding a backpressure of 9.39 × 10^3^ Pa at 0.5 μL/min. This approach avoids the significant cost in computational time that would be required if the full capillary lengths were simulated. A list of dimensions of components of the probe’s design is in Supporting Information section S4. The difference in viscosity of solution at body temperature and room temperature is significant, so viscosity and diffusion coefficients of peptides and solution conductivity have two values in Table 1.

### Laboratory

Probe Manufacture is Described in Supporting Information, S3.

#### Sample Preparation

A stock solution of 1.0 mM yaGfl (Shanghai Royobiotech, Shanghai, China) was prepared by diluting the solid in a modified Ringer’s solution consisting of 148 mM NaCl (EMD-Millipore, Darmstadt, Germany), 1.2 mM CaCl_2_ (EMD-Millipore), 2.7 mM KCl (Sigma-Aldrich), and 0.85 mM MgCl_2_ (Fisher Scientific, Fair Lawn, NJ) at pH 7.4. All solutions were then filtered using a 0.2 μm PES syringe filter (Corning Inc., Corning, NY) prior to use. To determine the limit of detection and linearity over relevant concentration ranges, a series of standards ranging from 100 to 1.56 μM were made through serial dilution.

#### Column Preparation

We used the Kasil method^72,73^ for making outlet frits for the 150 μm i.d. × 360 μm o.d. column blanks. A 25% (v/v) solution of formamide (Acros, NJ) and Milli-Q water was diluted 1:1 with potassium silicate (Kasil, PQ Corporation, Valley Forge, PA). A 10 μL drop was then placed on a piece of Whatman filter paper (Whatman, UK, catalog no. 1822-025). The end of the column blank was dipped on to filter paper and placed in a Thermo Focus series GC oven set to 85 °C for 12 h. The Acquity CSH C18 1.7 μm particles (Waters, Milford, MA) were slurried in 2-propanol at concentrations of 65 mg/mL. This was sonicated for 20 min before packing using the downward slurry method. A Haskel model DSF-150 pneumatic amplification pump (Burbank, CA) was used to pack the column at 20 000 psi for 20 min using methanol as the packing solvent before allowing the pressure to dissipate naturally. The column was trimmed to a final length of 10 cm.

#### Chromatography

The separation was achieved using a Dionex UltiMate 3000 Nano LC system (NCS-3200RS, Thermo Scientific, Germering, Germany) fitted with a micro-LC flow selector to deliver a mobile phase. Channel A contained 0.1% trifluoroacetic acid (TFA, Sigma Aldrich) in Optima LC-MS grade water (Fisher Chemical), and channel B contained 0.1% TFA in LC-MS grade acetonitrile (Fisher Chemical). Isocratic elution at 20% channel B was used at a constant flow rate of 2 μL/min for the entirety of the experiment. The pump was connected to an externally mounted 6-port two-position Cheminert injection valve (C72x-669D, VICI Valco, Houston, TX) using a 750 mm × 0.100 mm nanoViper capillary. A 140 mm × 0.100 mm nanoViper capillary was used as a 1.1 μL sample loop. A 350 mm × 0.025 mm i.d. × 0.360 mm o.d. fused silica capillary was used to connect the outlet of the column to a Waters Acquity TUV detector fitted with a 10 nL nano flow cell (Waters Corporation, Milford, MA) set to 214 nm. An Atlas analog-to-digital converter and Chromeleon version 6.8 software (Thermo) were used to acquire data at 100 Hz.

The yaGfl standards were injected in triplicate (21 data). The regression results from data (peak area vs injected concentration) indicated an intercept indistinguishable from 0 (95% CI of −0.0026 to +0.0049) and a slope of 0.00575 (95% CI of 0.00566 to 0.00583)

#### Preparation of the Device

The collection channel should be filled with modified Ringer’s solution prior to emplacing the microdialysis probe. To fill the collection channel, a 2 cm segment of 75 μm i.d. × 150 μm o.d. fused silica capillary was threaded into a 5 cm segment of 200 μm i.d. × 360 μm o.d. capillary. The interface was sealed with a drop of 2-ton epoxy (Devcon) and allowed to cure for 2 h at room temperature. The 75 μm i.d. × 150 μm o.d. segment was then cut to a length less than the height of the MD chamber (<1.35 mm). Finally, the 200 μm i.d. × 360 μm o.d. capillary was connected to a syringe using a Teflon union, and the 75 μm i.d. x 150 μm o.d. segment was inserted into the 290 μm diameter MD chamber. By applying force between the epoxy and the top surface of the MD sampling chamber, the chamber can be temporarily sealed to fill the collection channel with modified Ringer’s solution using the syringe. Microdialysis probes were constructed using hollow fiber dialysis membranes containing inlet and outlet fused silica capillaries (40 μm i.d., 100 μm o.d.). Capillaries were positioned with 1.0 mm vertical distance between the inlet and outlet capillary ends with an approximately 0.1 mm space between the outlet and the epoxy tip. The membranes are Spectra-Por RC Hollow Fiber; MWCO = 13 kDA, 200 μm i.d., 220 μm o.d. (Spectrum Laboratories Inc.; Rancho Dominguez, CA).

#### Animals

All procedures involving animals were approved by the Institutional Animal Care and Use Committee (IACUC) of the University of Pittsburgh. A male Sprague-Dawley rat (250–350 g, Hilltop, Scottsdale, PA) was anesthetized using isoflurane (5% induction, 2.5% maintenance) and placed in a stereotaxic frame (David Kopf Instruments, Tujunga, CA, USA). Animal placement was adjusted to flat skull, and the incisor bar was adjusted to reduce the dorsal measurement difference between lambda and bregma to less than 0.01 mm. The rat was wrapped in a heating blanket maintained at 37 °C. A minor craniotomy was performed over the prefrontal cortex (PFC), before lowering the device slowly (10 μm/s) into the PFC tissue (2.3 mm anterior and 3.0 mm lateral from bregma) to a final depth of 1.25 mm below dura. Aseptic technique was used throughout the experiment.

#### In Vivo Measurements

Prior to implantation, the EOP-EMD device and the microdialysis probe were soaked in 70% ethanol (Decon, King of Prussia, PA) for 20 min. One perfusion channel was loaded with the 1.0 mM yaGfl in modified Ringer’s solution, while the other channel was loaded with modified Ringer’s solution only. The MD chamber was filled with modified Ringer’s solution before inserting the MD probe. Using a Harvard Apparatus PHD 4400 programmable syringe pump (Holliston, MA), the MD probe was perfused with a modified Ringer’s solution at a flow rate of 0.5 μL/min. Before tissue implantation, the fluidic channels of the device were filled with modified Ringer’s solution and checked for the presence of bubbles. The device was lowered into a modified Ringer’s solution before connecting the silver electrodes in the perfusion channel and MD inlet to the current sources (model PS350, Stanford Research Systems Inc., Sunnyvale, CA). After 5 min of sustained current, the current was turned off and the device was lowered into the PFC. The yaGfl was infused with currents of (μA) 15, 30, 60, and 30 in that order. Chromatographic conditions are provided above. Data are shown in Supporting Information section S9.

## Supporting Information Available

Section S1 on overview of existing approaches to obtaining information about (ecto)peptidase activity; section S2 on schematic explanation of retrodialysis experiment to assess enzyme activity; section S3 on device creation; section S4 on dimensions used in the COMSOL model and Nanoscribe for the EOP-EMD probes; section S5 on pressure in tissue with 30 μA current flow rate/current relationship; section S6 on arrival time distribution from a pulse input; section S7 showing full view of the microdialysis chamber and the overflow exit; section S8 on derivation of flow rate per current relationship; section S9 showing experimental data and calibration slope/intercept/statistics (PDF)
