# Supplementary material for: Electroosmotic Perfusion, External Microdialysis: Simulation and Experiment
Source: ACS Chem Neurosci. 2023 Jun 28;14(14):2499–508. doi: 10.1021/acschemneuro.3c00057 (PMC10360060; doi:10.1021/acschemneuro.3c00057)
Supplement: Supplementary file 1 — cn3c00057_si_001.pdf [file cn3c00057_si_001.pdf]

SUPPORTING INFORMATION

Electroosmotic Perfusion - External Microdialysis: Simulation and Experiment

Michael T. Rerick, Jun Chen[†], Stephen G. Weber*
Department of Chemistry
Chevron Science Center
University of Pittsburgh
Pittsburgh PA 15260

[†]Petersen Institute of NanoScience and Engineering, and Department of Electrical and Computer Engineering
University of Pittsburgh
M104, Benedum Hall
3700 O'Hara Street, Pittsburgh, PA 15261

Electroosmotic Perfusion - External Microdialysis: Simulation and Experiment

Michael T. Rerick, Jun Chen, Stephen G. Weber*

SUPPORTING INFORMATION

1. S1 Overview of existing approaches to obtaining information about (ecto)peptidase activity.
2. S2 Schematic explanation of retrodialysis experiment to assess enzyme activity.
3. S3 Device creation.
4. S4 Dimensions used in the COMSOL model and Nanoscribe for the EOP-EMD probes.
5. S5 Pressure in tissue with 30 uA current Flow rate/Current relationship
6. S6 Arrival time distribution from a pulse input.
7. S7 Full view of the microdialysis chamber and the overflow exit
8. S8 Derivation of flow rate per current relationship.
9. S9 Experimental data and calibration slope/intercept/statistics.

S1. Overview of existing approaches to obtaining information about (ecto)peptidase activity

Artificial substrates are often used to assess enzyme activity. Elevated levels of matrix metalloproteases and other proteases [1] in the ECS are an indication of spreading cancer [2-6]. The methods used [7] are based on artificial substrates introduced into an animal with the goal of creating a signal or image indicating the presence of high enzyme activity, a characteristic of diseased tissue [1, 8, 9]. These methods usually acquire the signal or image from outside the animal/patient (fluorescence, near IR, PET, MRI) with clinical implications. In vitro measurements of relative changes or differences in ectopeptidase activity are common. Preparation of membrane fractions from tissue and use of artificial substrates, generally producing a fluorescent product, is a routine task, e.g.[10-17]. Imaging based on selective antibodies can also be used to identify the distribution of the enzyme among tissues [18]. Qualitative measurements of extracellular conversion of substrate to product have been made in vivo using artificial, fluorogenic substrates. Many investigators, e.g. [19], have used peptidase inhibitors in tissue or in vivo for identification of active enzymes. However, a recent review [20] on *in vivo* multifunctional devices with 434 citations contains zero citations to measuring enzyme reaction rates, peptidases, or proteases.

S2. Schematic explanation of retrodialysis experiment to assess enzyme activity

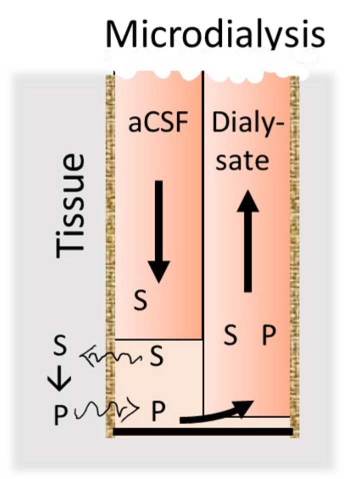

Fig. S1 Substrate, S, in artificial cerebrospinal fluid, aCSF, is introduced to tissue by diffusion through the membrane. Products, P, and remaining S are collected in dialysate.

S3 Device creation

The EOP-MD and EOP-EMD devices are compared in Figure S2 below.

The EOP-EMD device consists of six major components: (1) perfusion channels, (2) waste reservoir, (3) MD sampling chamber, (4) tip support, (5) source tip, and (6) the sink tip. These components are labeled in Figure S1. Two perfusion channels (1) combine to form one outlet at the perfusion tip (5). The horizontal distance between the perfusion and collection tip is 100 µm, corresponding to a distance of 269 µm between the center of each orifice. The outer diameter of both the perfusion and collection tip is 90 µm, with 50 µm internal channel diameters. A tip support (4) connects the upper portion of the perfusion and collection channels to prevent tip bending during the manufacturing and tissue implantation process. This tip support has a thickness of 10 µm, with the lower 0.1 mm forming a wedge to a final thickness of 1 µm. The conical portion terminating the perfusion and collection tips were designed to minimize penetration injury1 with an opening angle of 20°. The collection channel leads to a cylindrical MD sampling chamber (height = 1.35 mm, diameter = 290 µm). This MD sampling chamber also connects to a waste reservoir (88 nL) to prevent vacuum formation. The overall dimensions of the device used in the simulations and experiments are 0.965 mm (W) x 0.950 mm (L) x 3.71 mm (H).

The EOP-EMD device was printed with a 3D direct laser writing system (Nanoscribe Photonic Professional, GT). The COMSOL Multiphysics geometry was exported to Solidworks and saved into a standard stl file. The stl file was then loaded into the conversion software called 'DeScribe', where all the printing conditions can be defined. We chose a fixed slicing mode with a slicing distance of 0.8 µm, followed by a 'shell & scaffold' filling mode with a hatching distance of 0.5 µm, and scaffold spacing of 25 µm × 20 µm and wall line count of 1. Subsequently, the structure was split into blocks of 180 µm × 180 µm × 240 µm with a shear angle of 15 degree. A block overlap of 4 µm and layer overlap of 3 µm were chosen to compensate for potential drift from vibrations. The blocks were then set to be printed in a spiral order. After the conversion was done, the printing files were loaded into the operation software called 'Nanowrite'. A few droplets of IP-S resin were dispensed onto an ITO-coated glass substrate. The substrate was then loaded to Nanoscribe. Two-photon polymerization of the IP-S resist was used for printing by a femtosecond pulsed laser at 780 nm. A laser power of 90 mW and scan speed of 110 mm/s were used for the printing. After printing was completed, the substrate was unloaded and developed in the developer, 1-methoxy, 2-propanol acetate (PGMEA). A long development time of one to two hours was used to make sure the long and narrow channels were developed fully. Finally, the device was illuminated by a UV lamp (~ 16 mW/cm2) for an hour to solidify the whole structure.

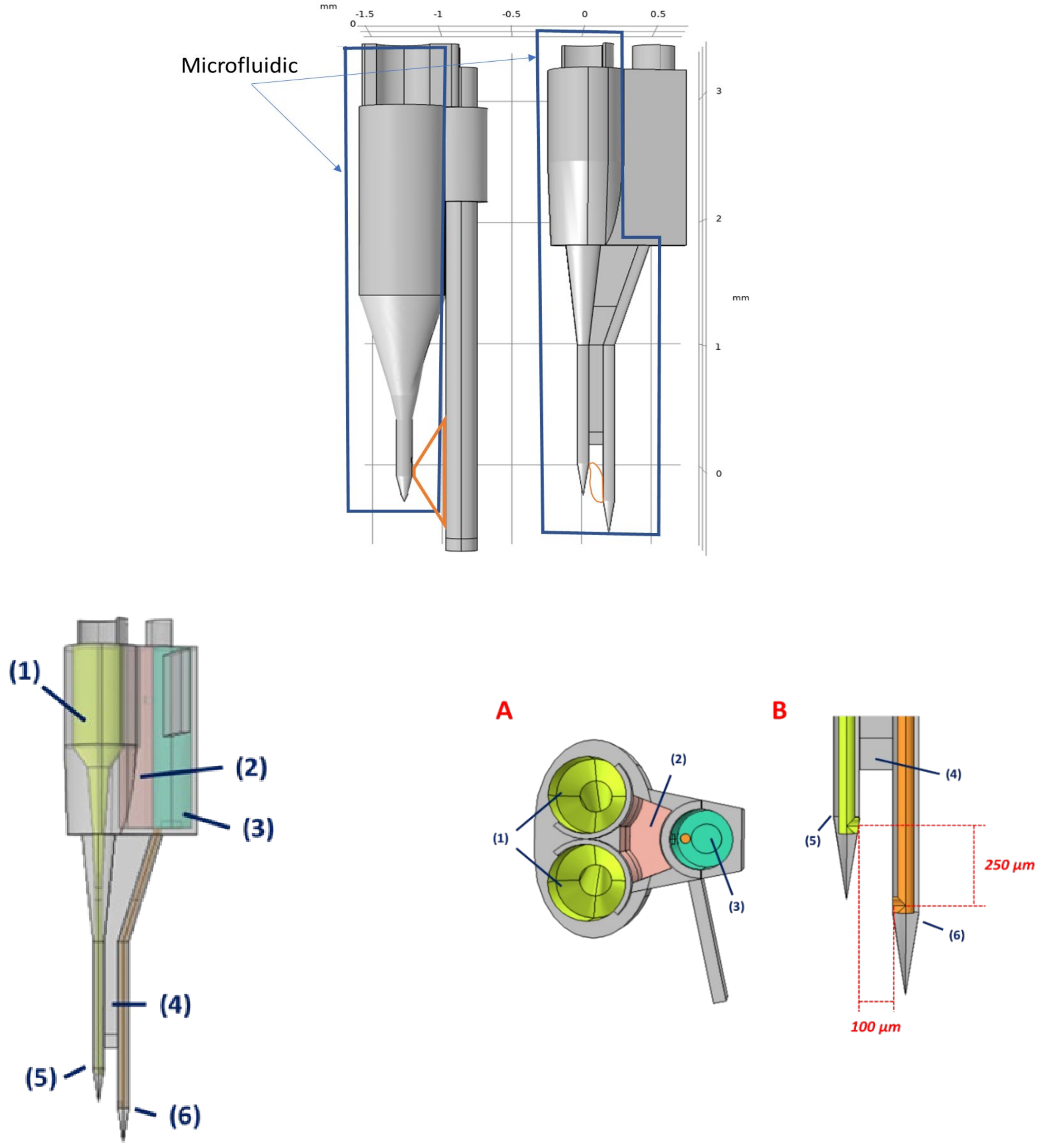

Fig. S2 Top Comparison of the gross anatomy of EOP-MD and EOP-EMD. Geometry of (left) EOP-MD and (right) EOP-EMD created within COMSOL Multiphysics. The left portions of each probe outlined in blue accept fused silica capillaries that carry the solution to be infused into the tissue. On the right-hand side of each is a microdialysis probe. It is evident in EOP-MD, but hidden in the EOP-EMD figure inside a chamber at the upper right. The orange-outlined regions approximately represent where tissue is sampled. **Bottom: Left** The fluidic channels consist of the perfusion channels (yellow), collection channel (orange), MD sampling chamber (cyan), and the waste reservoir (red). Numbers indicate functions: 1 – Perfusion channels, 2 – waste reservoir, 3 – MD sampling chamber, 4 – Tip support, 5 – Perfusion Tip, 6 – Collection tip. **Right**. More detail on EOP-EMD probe geometry. Numbers as in the previous image.

S4 Dimensions used in COMSOL model and manufacture of the EOP-EMD probes.

Description	Dimension
Center of MD probe	5.3E-4 m
Wall height separating upper channels	2E-4 m
Source tip eccentric cone length z	8E-4 m
Source eccentric cone angle	2.6E-4 m
Eccentric cone parameter	3.12
Capillary lumen area	4.4028E-9 m^2
ID of MD exit capillary (1/3)	4E-5 m
ID of MD exit capillary (2/3)	7.5E-5 m
ID of MD exit capillary (3/3)	2E-4 m
Capillary lumen area	1.2524E-9 m^2
Inner diameter of MD capillaries	4E-5 m
Outer diameter of MD capillaries	9.5E-5 m
Height of the MD Probe Housing	0.00135 m
MD Probe Housing ID	2.9E-4 m
Bottom of MD Housing Spacer height	5E-5 m
Bottom of MD Housing Spacer radius	7.5E-5 m
Length of MD inlet capillary od	1E-4 m
Effective length of MD membrane	0.001 m
Height of MD membrane	0.0012 m
Inner diameter of MD membrane	2E-4 m
Outer diameter of MD membrane	2.2E-4 m
Distance of outlet above the glue	1E-4 m
Support plate thickness	5E-5 m
Source capillary ID	1.56E-4 m
Source capillary inner height	5E-4 m
Source capillary upper od	3.7E-4 m
Distance between source channels	2E-5 m
Source tip y position	4.5E-5 m
Tip support cover x width	5E-5 m
Tip support cover y width	1.8E-4 m
Sampling tip ID	5E-5 m
Source tip x length	4E-5 m
Source Tip Length	0.001 m
Source tip channel radius	2.5E-5 m
Tip support length	7E-4 m
Tip taper length	1E-4 m
Height of tip in tissue	0.00135 m
X distance between source and collection tip	1E-4 m
Z offset between source and collection tip	2.5E-4 m
Length of tip point	1E-4 m
Height of tissue below tip	5E-4 m
Depth of Tissue	8E-4 m
Width of Tissue	0.002 m
Source channel xsec area	1.9635E-9 m^2

S5 Pressure within the tissue

Illustration of the pressure (Pa) within the tissue with 30 μA current. The vertical gradient (blue to green) in the extracellular space is due to gravity.

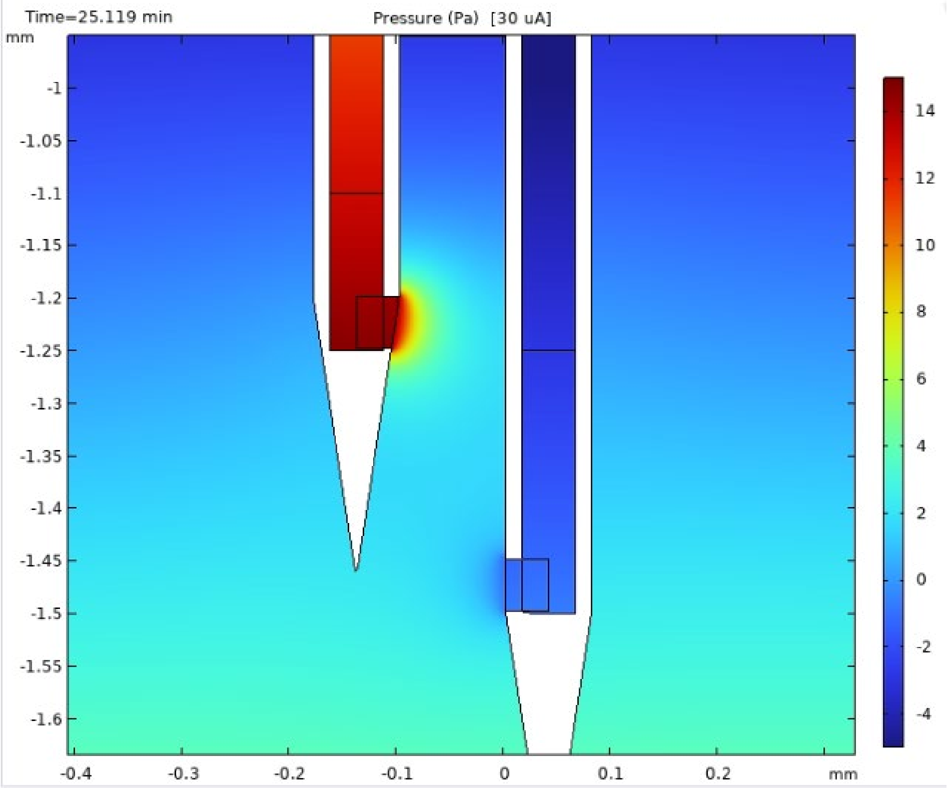

S6 Arrival time distribution from a pulse input. Note log time scale

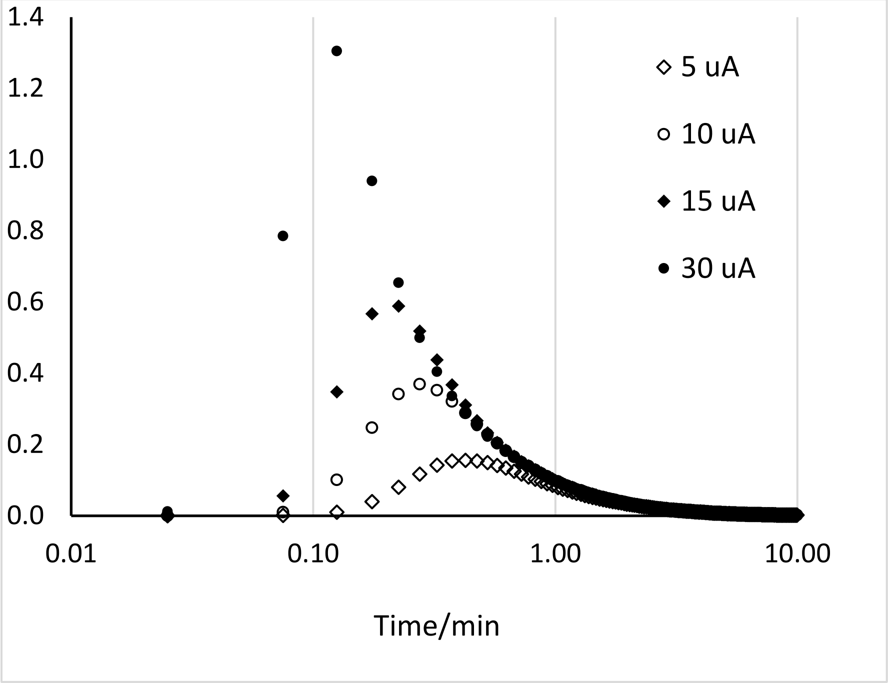

S7 Full view of the microdialysis chamber and the overflow exit (indicated as "5").

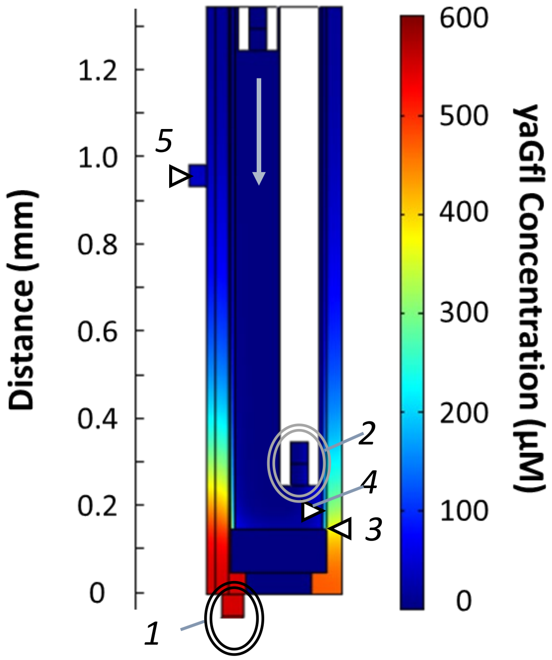

S8 Flow rate/Current relationship

The flow rate is velocity, v, times area, A:

$F = vA$ and $v = -\vec{E}\mu_{eo}$. $\vec{E}$ is electric field and μ_{eo} is electroosmotic mobility.

Further, with i current and ρ resistivity:

$\vec{E} = -\dfrac{i\rho}{A}$; thus $A = -\dfrac{i\rho}{\vec{E}}$.

Thus,

$$F = vA = (-\vec{E}\mu_{eo})(-\frac{i\rho}{\vec{E}}) = i\rho\mu_{eo}$$

Thus, $\dfrac{F}{i} = \rho\mu_{eo}$.

We use tortuosity λ =1.6 and porosity α = 0.2 from Sykova and Nicholson[52]. With a physiological saline conductivity G of 1.79 S/m then

$$\text{resistivity} = \frac{\lambda^2}{G\alpha} = 7.24\ \Omega m.$$

The electroosmotic mobility is $1.74\text{x}10^{-9}\ \dfrac{m^2}{Vs}$ in tissue. This is calculated as

$-\dfrac{\alpha}{\lambda^2}\dfrac{\varepsilon\varepsilon^o\zeta}{\eta}$ where $\varepsilon\varepsilon^o$ is water permittivity, ζ is the tissue zeta potential, and η is viscosity.

Finally:

$$\frac{F}{i} = \rho\mu_{eo} = 1.26\times10^{-8}\frac{m^3}{C} \approx 0.77\frac{nL}{\min\ \times\mu A}$$

S9 Experimental data

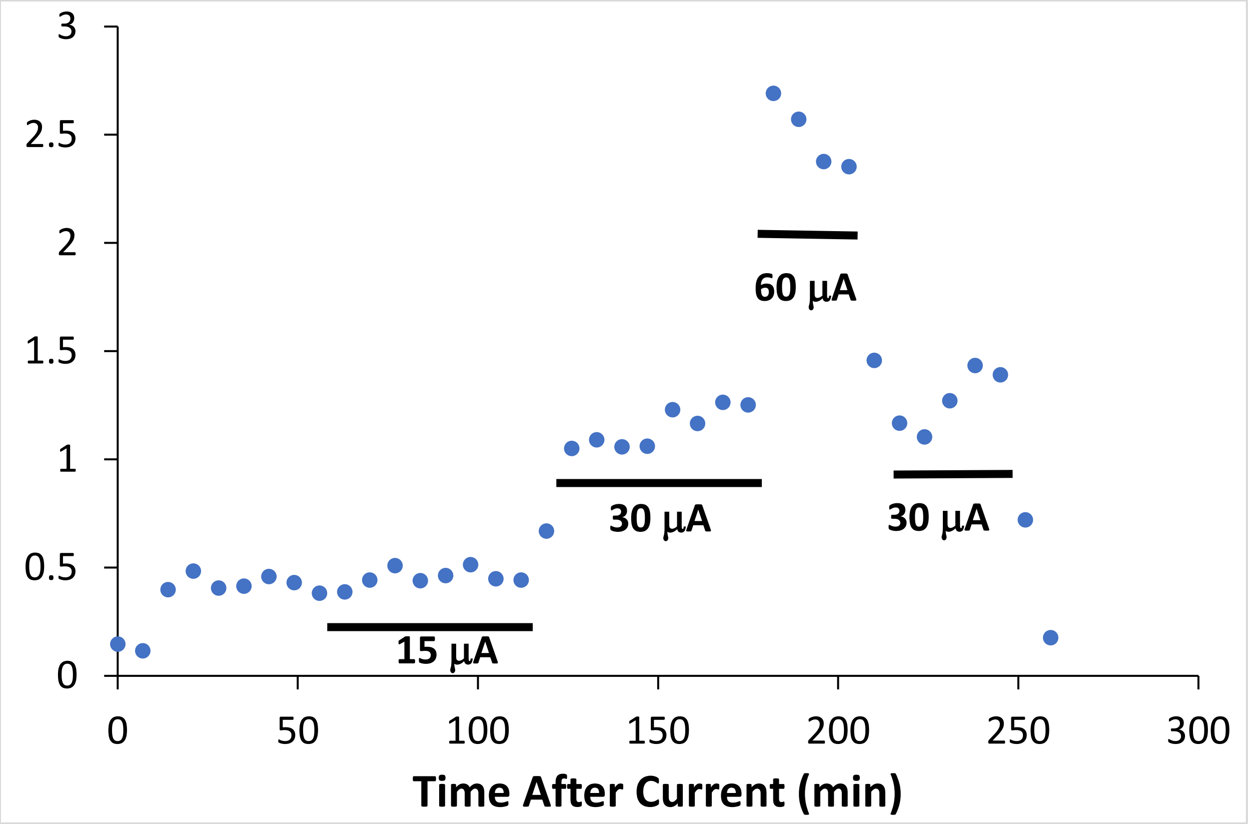

The plot shows the time course of measurements in vivo. Data taken for the plot shown in Fig. 8 of the main body of the manuscript are indicated with black underscore.

Calibration was carried out by LC-UV using serial 50% dilutions of yaGfl from 100 to 1.56 µM, with each concentration in triplicate (total 21 data). Regression (XLSTAT in Excel) showed an intercept statistically indistinguishable from 0 (0.001 with standard error 0.002, $t = 0.641$) and a slope 0.00575 ± 0.00009 (95% CI).

References

1. Soond, S. M., Kozhevnikova, M. V., and Zamyatnin Jr, A. A. (2019) 'Patchiness' and basic cancer research: unravelling the proteases, *Cell Cycle 18*, 1687-1701.
2. Ou, Y., and Weber, S. G. (2018) Higher Aminopeptidase Activity Determined by Electroosmotic Push-Pull Perfusion Contributes to Selective Vulnerability of the Hippocampal CA1 Region to Oxygen Glucose Deprivation, *ACS Chem. Neurosci. 9*, 535-544.
3. Krizkova, S., Zitka, O., Adam, V., Kizek, R., Masarik, M., Stiborova, M., Eckschlager, T., and Chavis, G. J. (2011) Assays for determination of matrix metalloproteinases and their activity, *TrAC, Trends Anal. Chem. 30*, 1819-1832.
4. Hu, B., Li, P., Zhang, Y., Shan, C., Su, P., Cao, J., Cheng, B., Wu, W., Liu, W., and Tang, Y. (2019) Activatable smart nanoprobe for sensitive endogenous MMP2 detection and fluorescence imaging-guided phototherapies, *Inorg. Chem. Front. 6*, 820-828.
5. Hu, B.-B., Li, P.-Y., Yang, X.-X., Fan, Y.-F., Shan, C.-F., Su, P.-R., Cao, J., Cheng, B., Liu, W.-S., and Tang, Y. (2019) Smart MMP2-Responsive Nanoprobe for Activatable Fluorescence Imaging-Guided Local Triple-Combination Therapies with Single Light, *ACS Appl. Bio Mater. 2*, 2978-2987.
6. Ceylan, H., Yasa, I. C., Yasa, O., Tabak, A. F., Giltinan, J., and Sitti, M. (2019) 3D-Printed Biodegradable Microswimmer for Theranostic Cargo Delivery and Release, *ACS Nano 13*, 3353-3362.
7. Ou, Y., Wilson, R. E., and Weber, S. G. (2018) Methods of Measuring Enzyme Activity Ex Vivo and In Vivo, *Annual Review of Analytical Chemistry 11*, 509-533.
8. R. Y. Suaifan, G. A., Jaber, D., Shehadeh, M. B., and Zourob, M. (2017) Proteinases as Biomarkers in Breast Cancer Prognosis and Diagnosis, *Mini-Rev. Med. Chem. 17*, 583-592.
9. Hillebrand, L. E., and Reinheckel, T. (2019) Impact of proteolysis on cancer stem cell functions, *Biochimie 166*, 214-222.
10. Gallegos, M. E. H., Zannatha, M. M. I., Osornio, E. G., Sanchez, A. S., and Del Rio, F. A. P. (1999) The activities of six exo- and endopeptidases in the substantia nigra, neostriatum, and cortex of the rat brain, *Neurochem. Res. 24*, 1557-1561.
11. Irazusta, J., Larrinaga, G., Agirregoitia, N., Varona, A., and Casis, L. (2003) Effects of morphine administration and its withdrawal on rat brain aminopeptidase activities, *Regul. Pept. 110*, 225-230.
12. Minnasch, P., Yamamoto, Y., Ohkubo, I., and Nishi, K. (2003) Demonstration of puromycin-sensitive alanyl aminopeptidase in Alzheimer disease brain, *Leg. Med. 5*, S285-S287.
13. Banegas, I., Prieto, I., Alba, F., Vives, F., Araque, A., Segarra, A. B., Duran, R., de Gasparo, M., and Ramirez, M. (2005) Angiotensinase activity is asymmetrically distributed in the amygdala, hippocampus and prefrontal cortex of the rat, *Behav. Brain Res. 156*, 321-326.
14. Larrinaga, G., Callado, L. F., Agirregoitia, N., Varona, A., and Gil, J. (2005) Subcellular distribution of membrane-bound aminopeptidases in the human and rat brain, *Neurosci. Lett. 383*, 136-140.
15. de Gortari, P., Angel Vargas, M., Martinez, A., I García-Vázquez, A., María Uribe, R., Chávez-Gutiérrez, L., Magdaleno-Madrigal, V., Boileau, G., Charli, j.-l., and Joseph-Bravo, P. (2007) *Stage-specific Modulation of Neprilysin and Aminopeptidase N in the Limbic System During Kindling Progression*, Vol. 33.
16. Hernandez, J., Segarra, A. B., Ramirez, M., Banegas, I., de Gasparo, M., Alba, F., Vives, F., Duran, R., and Prieto, I. (2009) Stress Influences Brain Enkephalinase, Oxytocinase and Angiotensinase Activities: A New Hypothesis, *Neuropsychobiology 59*, 184-189.
17. Morales-Mulia, M., Gortari, P., Amaya, M.-I., and Mendez, M. (2012) Activity and Expression of Enkephalinase and Aminopeptidase N in Regions of the Mesocorticolimbic System are Selectively Modified by Acute Ethanol Administration, *J. Mol. Neurosci. 46*, 58-67.
18. Goto, Y., Nakamura, T. J., Ogawa, K., Hattori, A., and Tsujimoto, M. (2020) Reciprocal expression patterns of placental leucine aminopeptidase/insulin-regulated aminopeptidase and vasopressin in the murine brain, *Front. Mol. Biosci. 7*, 168.
19. Littlewood, G. M., Iversen, L. L., and Turner, A. J. (1987) The effect of peptidase inhibitors on the recovery of substance P released from brain slices, *Biochem. Soc. Trans. 15*, 894-895.

20. Sung, C., Jeon, W., Nam, K. S., Kim, Y., Butt, H., and Park, S. (2020) Multimaterial and multifunctional neural interfaces: from surface-type and implantable electrodes to fiber-based devices, *J. Mater. Chem. B 8*, 6624-6666.
